# Defining the mid-diastolic imaging period for cardiac CT – lessons from tissue Doppler echocardiography

**DOI:** 10.1186/1471-2342-13-5

**Published:** 2013-02-01

**Authors:** James M Otton, Justin Phan, Michael Feneley, Chung-yao Yu, Neville Sammel, Jane McCrohon

**Affiliations:** 1 Cardiology Department, St Vincent’s Hospital, Darlinghurst, Sydney, 2010, AUSTRALIA; 2 University of New South Wales, Sydney, NSW, Australia

**Keywords:** Cardiac CT, Image quality, Heart rate, Tissue Doppler, Echocardiography

## Abstract

**Background:**

Aggressive dose reduction strategies for cardiac CT require the prospective selection of limited cardiac phases. At lower heart rates, the period of mid-diastole is typically selected for image acquisition. We aimed to identify the effect of heart rate on the optimal CT acquisition phase within the period of mid-diastole.

**Methods:**

We utilized high temporal resolution tissue Doppler to precisely measure coronary motion within diastole. Tissue-Doppler waveforms of the myocardium corresponding to the location of the circumflex artery (100 patients) and mid-right coronary arteries (50 patients) and the duration and timing of coronary motion were measured. Using regression analysis an equation was derived for the timing of the period of minimal coronary motion within the RR interval. In a validation set of 50 clinical cardiac CT examinations, we assessed coronary motion artifact and the effect of using a mid-diastolic imaging target that was adjusted according to heart rate vs a fixed 75% phase target.

**Results:**

Tissue Doppler analysis shows the period of minimal cardiac motion suitable for CT imaging decreases almost linearly as the RR interval decreases, becoming extinguished at an average heart rate of 91 bpm for the circumflex (LCX) and 78 bpm for the right coronary artery (RCA). The optimal imaging phase has a strong linear relationship with RR duration (R^2^ = 0.92 LCX, 0.89 RCA). The optimal phase predicted by regression analysis of the tissue-Doppler waveforms increases from 74% at a heart rate of 55 bpm to 77% at 75 bpm. In the clinical CT validation set, the optimal CT acquisition phase similarly occurred later with increasing heart rate. When the selected cardiac phase was adjusted according to heart rate the result was closer to the optimal phase than using a fixed 75% phase. While this effect was statistically significant (p < 0.01 RCA/LCx), the mean effect of heart-rate adjustment was minor relative to typical beat-to-beat variability and available precision of clinical phase selection.

**Conclusion:**

High temporal resolution imaging of coronary motion can be used to predict the optimal acquisition phase in cardiac CT. The optimal phase for cardiac CT imaging within mid-diastole increases with increasing heart rate although the magnitude of change is small.

## Background

Improvements in cardiac computed tomography technology have enabled fast and reliable imaging of the coronary arteries. Nevertheless, temporal resolution remains a limiting factor and the timing of image acquisition is critical for optimal imaging quality. While various empiric principles have been adopted to guide coronary image acquisition, to date, studies of timing strategies have generally focused on preferable timing bands of CT acquisition [1-4] during systole or diastole according to heart rate. Mid-diastole is typically selected at lower heart rates. Precise guidance as to the optimal phase within this period has been limited due to the temporal resolution of coronary tracking techniques available.

Echocardiography, and in particular tissue Doppler, is particularly suited due to its high temporal resolution, accuracy and ability to interrogate the myocardial motion at the arterial segments most vulnerable to imaging artifact [5]. Tissue Doppler provides motion assessment of the coronary arteries with a temporal resolution of less than 5 ms [6] as opposed to the much poorer 30-50 ms resolution of invasive angiography [7,8], 10-20 ms resolution for MRI [9] or the 70-160 ms temporal resolution of CT imaging itself [10]. Tissue Doppler may therefore be used to distinguish between coronary motion present at single-percentage phase intervals within the diastolic period.

Optimal timing of cardiac CT is important firstly to minimize or eliminate motion artifact, and secondly to guide imaging parameters with regards to aggressive dose minimization strategies, which frequently employ limited cardiac phases [11,12]. It is well recognized that the fraction of the cardiac cycle occupied by diastole decreases with increasing heart rate and comes proportionally later in the cardiac cycle. It would therefore be expected that the optimal window for cardiac imaging may vary with heart rate and that small adjustments to the acquired phase may result in improved image quality.

We sought to investigate the motion of the coronary arteries throughout the cardiac cycle in order to provide both a qualitative and mathematical description of the period of optimal diastolic cardiac image acquisition in order to guide coronary imaging by low-dose, limited-phase cardiac CT. We compared the resultant analysis and prediction equations to motion artifact outcomes within a clinical cardiac CT cohort.

## Methods

Echocardiographic measurements were obtained from anonymised routine clinical data and participants within the CT cohort gave written consent for the use of clinical data for research purposes. The study was performed in accordance with the principles of the Helsinki declaration and was approved by the St Vincent’s Hospital Human Ethics Committee (approval number 10/044).

In order to record physiological motion of the coronary arteries, consecutive clinical echocardiograms performed at our institution were retrospectively reviewed from June 2010. Echocardiograms were performed using either a Philips ie33 or Sonos 7500 echocardiography system by an experienced technician. One hundred echocardiograms with clear tissue Doppler traces of the left ventricular lateral wall and fifty with right ventricular lateral wall tissue Doppler were selected from a sequential cohort of 324 patients. Patients were referred from both inpatient and outpatient settings for standard indications including the assessment of ischemic heart disease, heart failure and valvular pathology. Exclusion criteria were poor image quality and the presence of a cardiac rhythm other than sinus rhythm.

The tracked volume was selected to encompass the lateral tricuspid annulus corresponding to the course of the mid right coronary artery and the lateral mitral valve annulus, corresponding to the mid-distal position of the circumflex artery.

**Figure 1:**
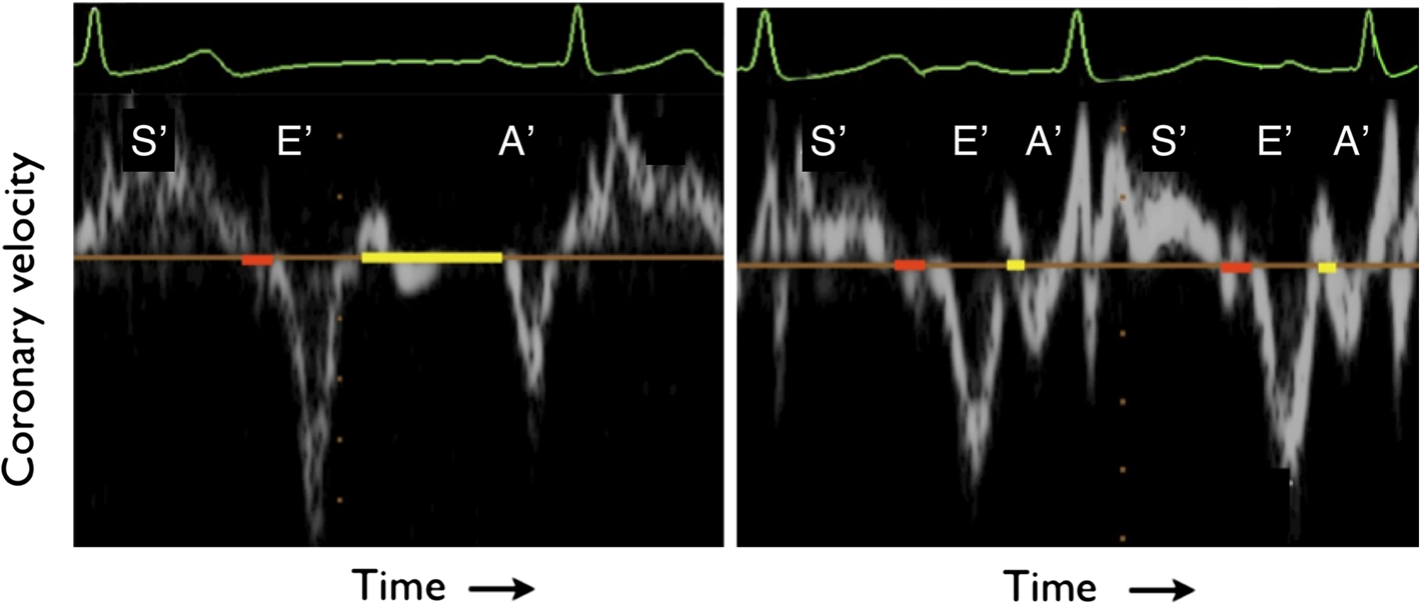
**Tissue Doppler waveforms of the left atrio-ventricular groove (circumflex artery) at low (left) and high (right) heart rate.** Coronary motion occurs throughout systole (S’). At the end of systole there is a short period of relative stasis between the closing of the aortic valve and the opening of the mitral valve (isovolumic relaxation time, IVRT), shown in red. This period varies little with heart rate. The period of minimal cardiac motion (yellow band) occurs between the E’ and A’ waves. The PMCM shortens with increasing heart rate. Images have been resized and edited for clarity.

Tissue Doppler provides the motion of the area of tissue selected, relative to the transducer, typically at approximately 4 ms time intervals with the velocity displayed as per the left hand scale in cm/s. The tissue Doppler signal (Figure 1) is formed initially by the S’ wave, corresponding to ventricular systole, a short period of little to no movement reflecting the isovolumic relaxation time (IVRT) and shift from systolic to diastolic phases. The E’ corresponds to rapid ventricular filling due to ventricular relaxation. A short period of almost no cardiac motion then follows at low heart rates- the period of minimal cardiac motion (PMCM)- and then the A’ wave reflective of atrial contraction. This PMCM is indicative of the ideal period of mid-diastolic coronary CT acquisition.

Utilizing the recorded ECG trace simultaneous to echocardiographic image acquisition, the time interval from the mid-point of the QRS trace to the start and end of the isovolumic relaxation time (IVRT), center of the E’ and A’ waves and start and end of the period minimal cardiac motion (PMCM) was measured. The period of minimal cardiac motion was defined as the diastolic phase between the E’ and A’ during which time the spectrum of the tissue Doppler velocity trace indicated tissue velocity of less than 2 cm/s. The duration of the QRS interval, current and preceding RR interval and ejection fraction were also noted.

### Clinical CT validation study

In a validation cohort of 50 consecutive cardiac CT studies we prospectively assessed motion artifact within mid-diastole. The prospective cohort of 50 consecutive clinical cardiac CT patients included all scans performed for standard clinical indications, and included a broad range of heart rates. Patients were excluded from the validation cohort if the underlying rhythm was not sinus rhythm. Patients within the derivation echocardiography set were not included within the CT validation cohort.

All CT scans were obtained utilizing a 320 detector-row CT (Toshiba Aquilion One) with z-axis coverage adjusted to ensure complete cardiac coverage within a single gantry rotation and image acquisition of the entire coronary tree at identical cardiac phase. The x-ray tube current (mA), voltage (kV) and contrast volume (Ultravist370) were adjusted according to body size and standard clinical protocols. A FC03 filter kernel was used for reconstruction, without the use of iterative noise reduction. The resultant CT images were displayed at 0.5 mm [3] isotropic resolution. Where multiple cardiac cycles were acquired (for example due to very high heart rates or multiple volume acquisition), only the first RR interval was used for timing analysis.

The acquired cardiac phase was defined as the percentage of the RR interval corresponding to the midpoint of the half-scan reconstruction. All scans were acquired with a minimum of 170 ms of widened data acquisition, enabling reconstruction of a range of 70-80% of cardiac phase or greater, during which the mid-diastolic period is expected to occur at all physiological heart rates. In patients with a baseline heart rate of greater than 65 beats per minute or greater, a 30-80% range of cardiac phases were acquired. In all scans it was anticipated that the period of mid-diastole would be fully captured.

**Figure 2:**
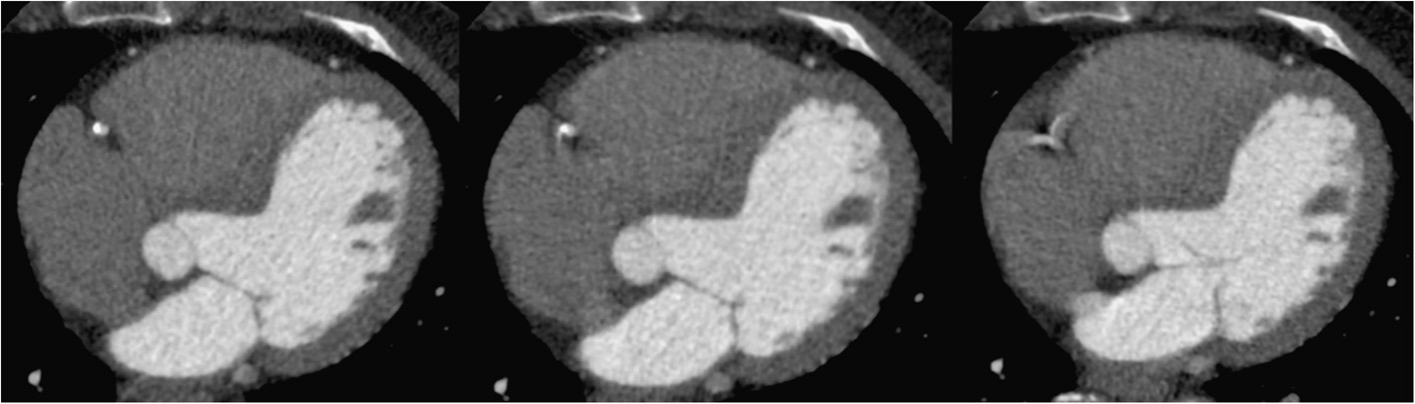
**Examples of motion artifact definitions at the mid-right coronary artery.** (Left) No artifact (Middle) Mild artifact (Right) Severe motion artifact. Phases containing no motion artifact were assigned to the period ideal cardiac imaging.

Motion artifact was defined as any blurring or loss of clear delineation of the coronary lumen and was assessed by a single reader (Figure 2). The cardiac phases at which motion artifact was absent was noted at both the mid right coronary artery and within the left coronary circulation at the bifurcation of the proximal left anterior descending artery and circumflex arteries. The ideal imaging target was selected separately for each artery as the mid-point between the first and last phases of artifact-free images. Where no motion free phase occurred, the phase containing the least motion artifact was selected as the ideal imaging target.

### Statistical methods

#### Tissue Doppler analysis

Utilizing separate ordinary least squared linear regression models within STATA statistical software v10, StataCorp, USA we assessed the relationship between the RR interval and the dependent variables: time to the center of the IVRT, time to the E’ wave, time to the A’ wave and time to center of the period of minimal cardiac motion. The relationship and regression residuals were inspected visually. In addition, non-linearity was assessed using the Ramsay RESET test for omitted variables. Using simple algebraic substitution (noting Heart Rate = 60 seconds/RR interval) the regression equation relating the RR interval and the time to the center of the PMCM was used to form a prediction equation relating heart rate to the ideal target of cardiac imaging. This heart rate adjusted ideal phase equation was subsequently tested in the CT validation cohort.

As the duration of the PMCM, or imaging window, is limited by a lower bound of zero, truncated linear regression was used to generate an equation relating the RR interval and PMCM. This equation was used to forecast the PMCM based on the RR interval. The standard deviation of the forecast was calculated and used to create 90% confidence intervals of the forecast, which was then graphically displayed. The forecast equation of the PMCM was then displayed against heart rate within heart rates clinically relevant to diastolic imaging. This allowed the relationship between the CT acquisition time and the probability that the acquisition time falls within the PMCM to be graphically demonstrated.

#### CT validation cohort

The relationship between heart rate and ideal imaging target (the mid-point of the artifact free CT image time period) was assessed using ordinary least square regression. The null hypothesis, that there was no relationship between heart rate and ideal imaging target, was tested using a two-sided T test of the regression slope coefficient.

The timing error, or the absolute difference in time between the ideal imaging phase as judged by CT and

a) A fixed 75% phase

b) The heart-rate predicted ideal imaging phase

was measured. The error of each method of phase selection was compared using a paired t-test. The two-sided threshold for significance of the T-test was set at p < 0.05.

## Results

### Timing of tissue Doppler features and the cardiac imaging window

**Figure 3:**
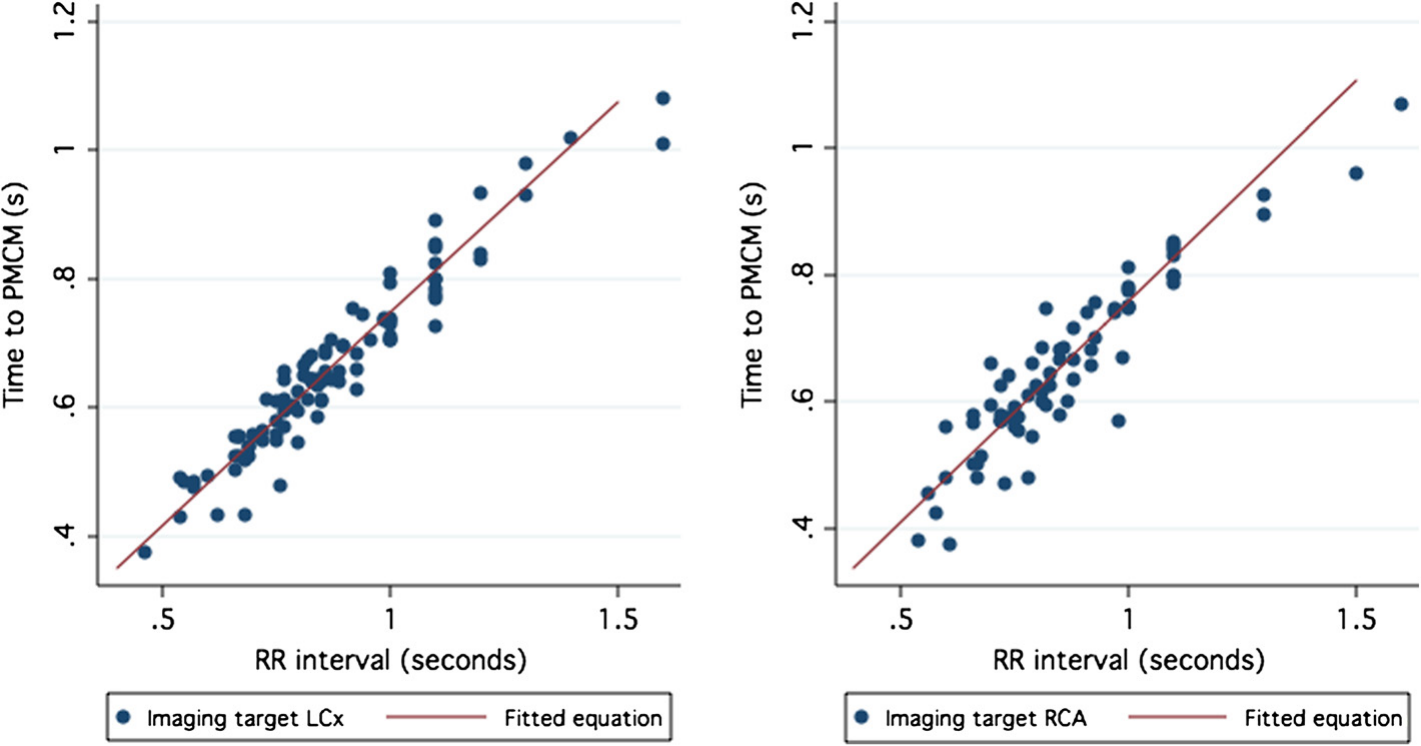
**Time to ideal imaging target accord to the RR interval for the Circumflex (left) and Right Coronary (right) arteries.** The fitted regression equation is also illustrated.

**Figure 4:**
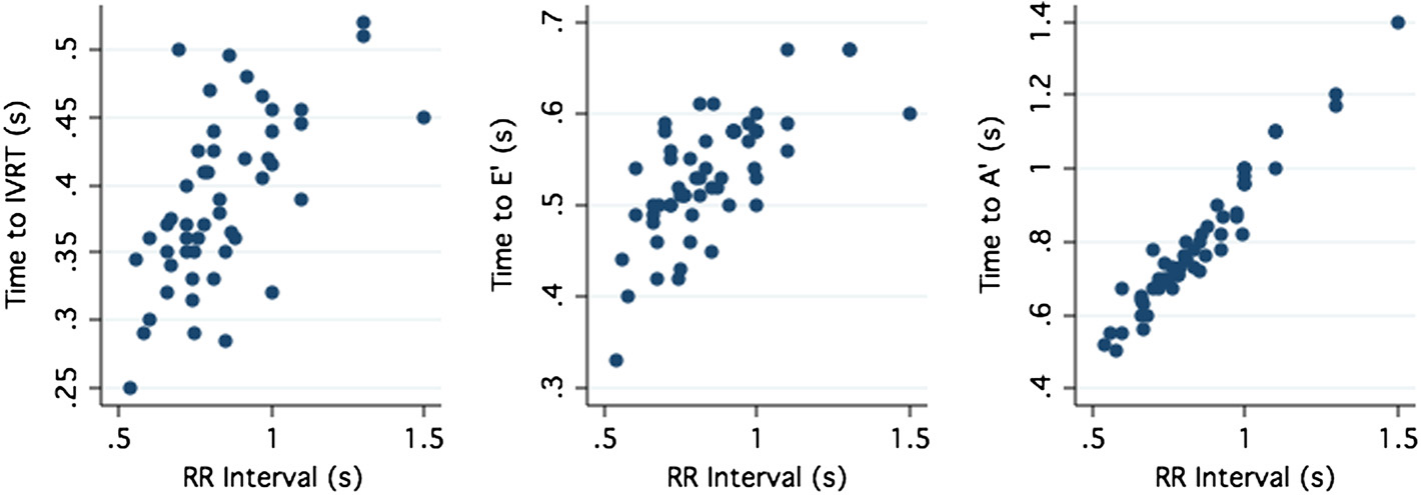
Time interval to the IVRT (Left), E’ wave (Middle) and A’ wave (Right) according to RR interval.

Visual examination of the relationship between the RR interval and the time to the E’ wave, time to the A’ wave, time to the IVRT and time to the center of the PMCM for the circumflex and right coronary arteries (Figures 3 and 4) indicated that the relationship between the variables did not demonstrate evident non-linearity, except at very low heart rates. This finding was mirrored by examination of the regression residuals. When heart rates < 50 where excluded, the Ramsay RESET test for omitted variables, indicative of non-linearity, were non-significant (p > 0.3) for all variables.

The center of the right coronary IVRT showed only a modest correlation with heart rate (R^2^ = 0.38) irrespective of QRS duration or ejection fraction. The center of the IVRT occurred within the range (95% confidence interval) 32–63% of the RR duration, occurring later as a percentage of the RR interval as the heart rate increased

Correlation of the time to E’ was strongly related to the RR duration (LCX R^2^ = 0.60; RCA R^2^ = 0.52 p < 0.001 for both). The A’ was very strongly related to the RR duration (LCX R^2^ = 0.96; RCA R^2^ = 0.92. P < 0.001 for both). (Figure 4).

The coefficient of determination (R^2^) of the relationship between the ideal imaging target and the RR interval was 0.92 for the circumflex and 0.86 for the right coronary artery. The regression equation for the optimal target of CT acquisition was found to be:

Optimal CT trigger time(ms) Circumflex = RR interval x 0.67 + 80 ms.

Optimal CT trigger time(ms) Right Coronary Artery = RR interval x 0.68 + 70 ms.

Heart rates outside the clinical range for diastolic imaging, 50–90 bpm, were excluded due to the excessive leverage of outlying values on regression analysis. The resultant heart rate targets, which are the same for both the circumflex and right coronary arteries are shown in Table 1.

**Table 1:** Heart rate adjusted phase targets for both the right and circumflex coronary arteries

									
Heart Rate	50	55	60	65	70	75	80	85	90
Optimal diastolic target (% phase)	74%	74%	75%	76%	76%	77%	78%	78%	79%

### Doppler study of the duration of the cardiac imaging window in mid-diastole

As expected, tissue Doppler recording of both the right and left atrio-ventricular groove demonstrated greatest motion during systole. The mean duration of the IVRT, or end-systolic imaging window, was 55 ms and only a weak positive relation with the RR interval was noted (R^2^ = 0.28, p < 0.001).

**Figure 5:**
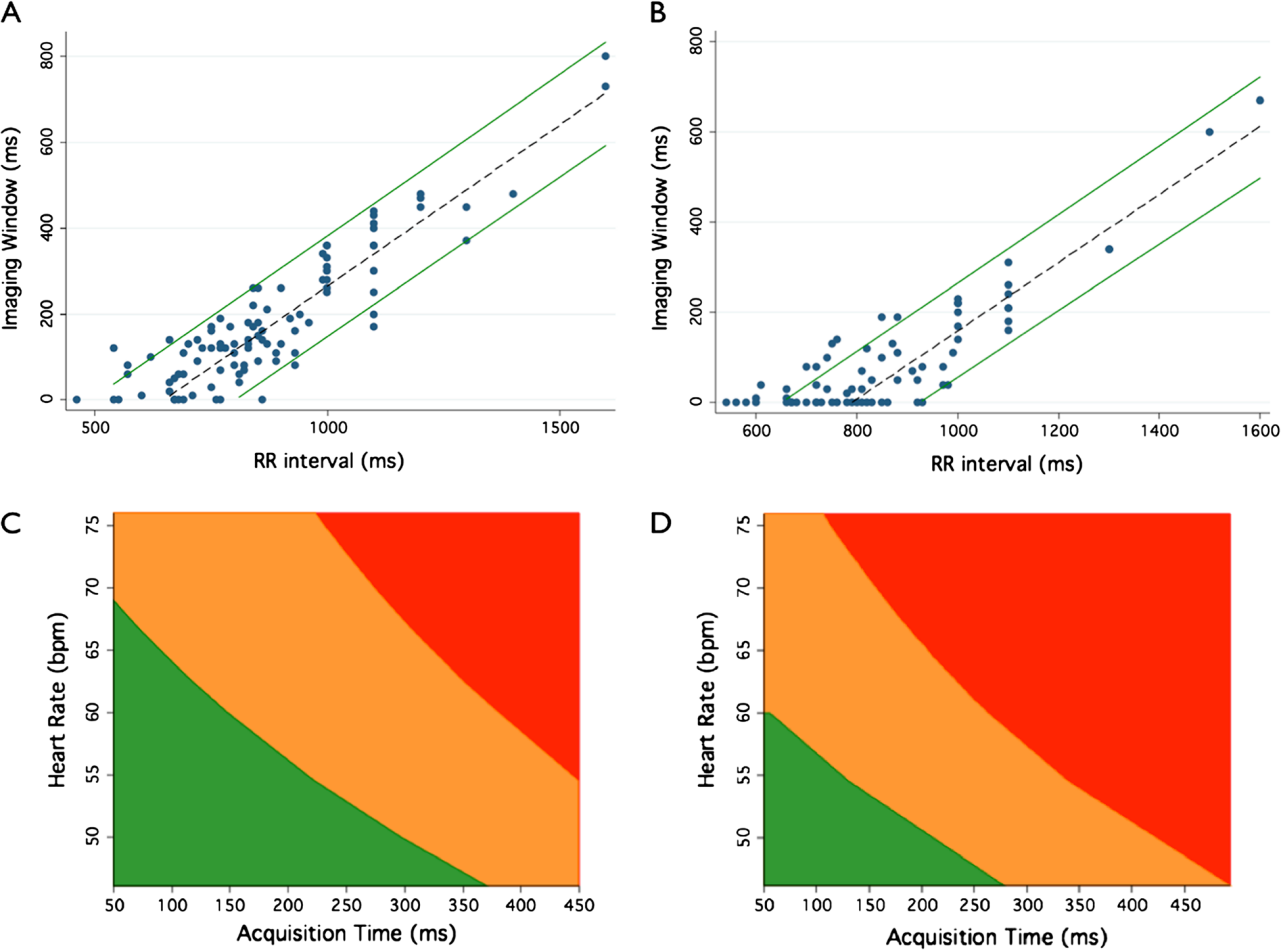
**The duration of minimal cardiac motion suitable for cardiac imaging as assessed by tissue Doppler.** The PMCM increases with the RR interval for both the circumflex (**A**) and right (**B**) coronary arteries, shown with 90% forecast intervals. The confidence intervals of graph (**A**) may be transformed to form a guide to the likelihood of imaging artifact in the circumflex (**C**) and right coronary arteries (**D**) based on heart rate and acquisition time (half gantry rotation time). Within the green area, less than 5% of coronary arteries are expected have any motion artifact. Motion artifact may occur within the amber zone (> 5% chance of artifact). The red area reflects a very high (> 95%) probability of motion artifact.

By contrast, the duration of diastolic PMCM is highly dependent on the heart rate extending almost linearly with increased RR interval (Figure 5A,B). At shorter RR intervals, the PMCM becomes progressively shorter, approaching zero above heart rates averaging 91 bpm for the circumflex and 78 bpm for the right coronary artery.

**Figure 6:**
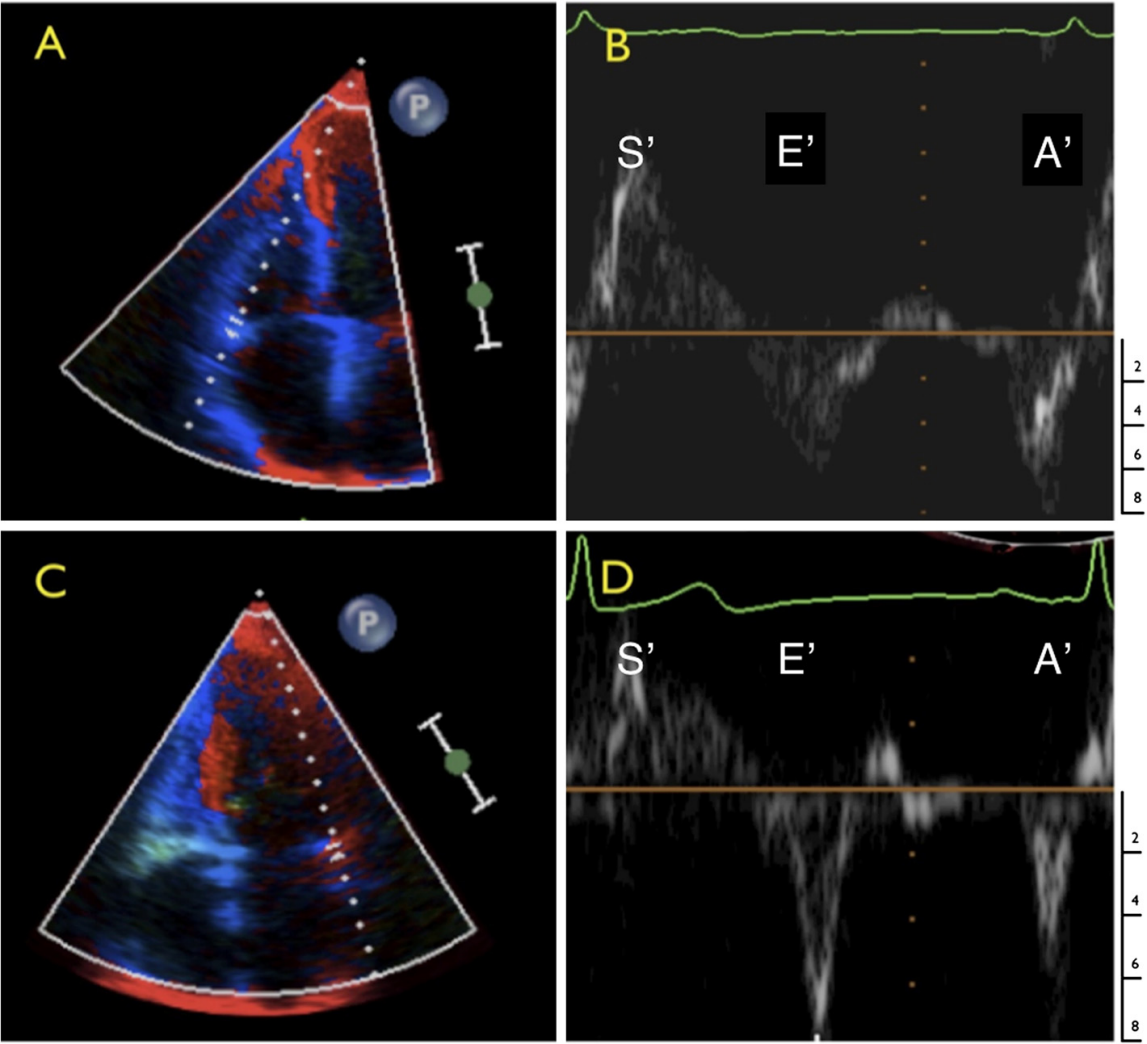
**Tissue Doppler waveforms of the right (A) and left (C) atrio-ventricular grooves (Circumflex and Right coronary arteries).** The right coronary artery E’ and A’ waves (**B**) tend to be broader leaving a shorter period of minimal cardiac motion. While the magnitude of each wave varies between individuals, the integral of the wave, or total motion is usually greater for the right than circumflex coronary arteries. This is the corollary of the rapid and more prolonged period of motion of the right coronary artery as may be seen during cine coronary angiography [7]. The duration of the PMCM of the right coronary between the E’ and A’ is also reduced relative to the Circumflex artery (**D**). Shown with indicative scale (cm/s). S’ indicates systolic myocardial velocity.

The duration of the circumflex PMCM averages 260 ms at a heart rate of 60, 150 ms at a heart rate of 70 and 77 ms at a heart rate of 80 bpm. The right sided PMCM is of shorter duration (Figure 6), reflecting the narrower imaging window and resultant greater chance of imaging motion artifact as illustrated in Figure 5C and D.

### CT validation

Basic demographic details of patients studied are presented in Table 2. Ninety percent of patients received a rate controlling medication to achieve a target heart rate of 65 beats per minute or lower. 82% received oral beta-blockers, 4% IV beta-blockers and 2% received ivabradine or diltiazem respectively. Metoprolol was used in the large majority of cases and the median beta-blocker dose was 75 mg (range 25-200 mg). Due to extensive rate control, the median heart rate was 60 and only 3 patients recorded a heart rate of greater than 75 at the time of the CT scan.

**Table 2:** Validation patient demographic data

**Variable**	**Median(Range)**
Age, years	61 (24–75)
Weight (kg)	80 (44–135)
Body Mass Index	27 (18–37)
Heart rate	60 (43–102)

In 38% of patients the stated reason for referral for CT was assessment due to cardiovascular risk factors or equivocal prior investigations. In 34% of patients the stated reason for referral for cardiac CT was of chest pain, and 14% were referred for prior arrhythmia. No referral reason was recorded for 14% of patients. The median radiation dose of all scans was 6.2 mSv.

The ideal cardiac phase for both the right and left coronary arteries increased with heart rate. The regression coefficient for the circumflex was 0.17 (95% CI 0.090-0.25, T-test P < 0.01) and 0.15 (95% CI 0.038-0.27, T-test P < 0.05) for right coronary artery indicating a 1.7% or 1.5% increase in the ideal imaging target in mid-diastole for every 10 beat increase in heart rate.

Analysis of time between the ideal cardiac imaging target, 75% phase target and the heart rate adjusted target (as per Table 1) demonstrated that the heart rate adjusted target was statistically closer to the ideal target than the fixed 75% target for both the right coronary artery and left anterior descending/circumflex arteries, (p < 0.01 for both). The absolute benefit of the heart rate adjusted strategy was 0.5% (SD 0.14%) for the circumflex and 0.4% (SD 0.13%)for the RCA. This partially reflects minor differences between the two prediction models at the most common heart rate 55–65 bpm. At heart rates greater than 65 the heart rate adjusted strategy was a mean of 1% for the RCA and 0.6% for the circumflex, closer to the ideal imaging phase.

**Figure 7:**
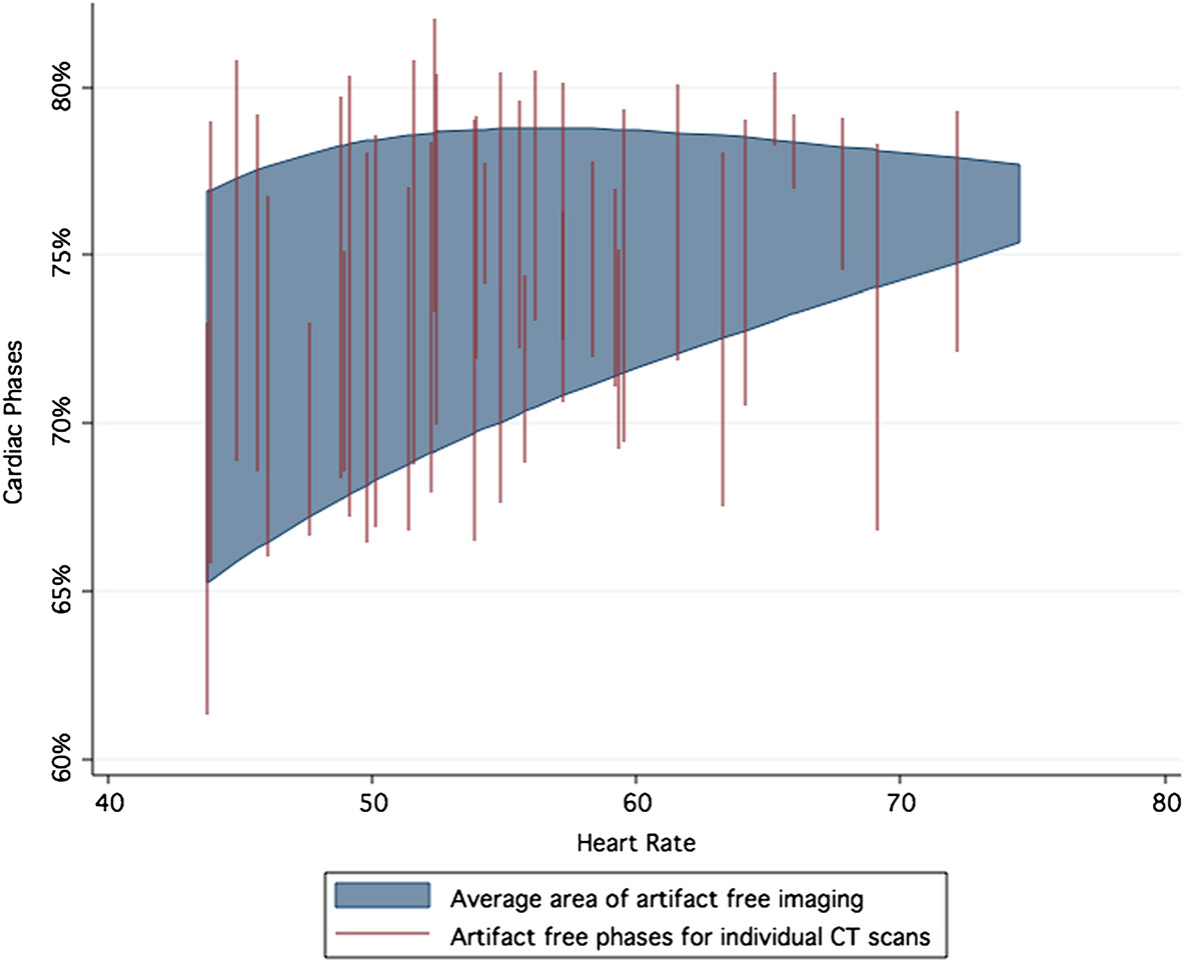
**Motion-free acquisition windows for the circumflex artery from the CT validation set.** Substantial variation in the motion-free period exists between individual studies. The fitted average of the motion-free period is shaded. While the upper bound of the area remains relatively constant with heart rate, the lower bound increases dramatically with heart rate.

**Figure 8:**
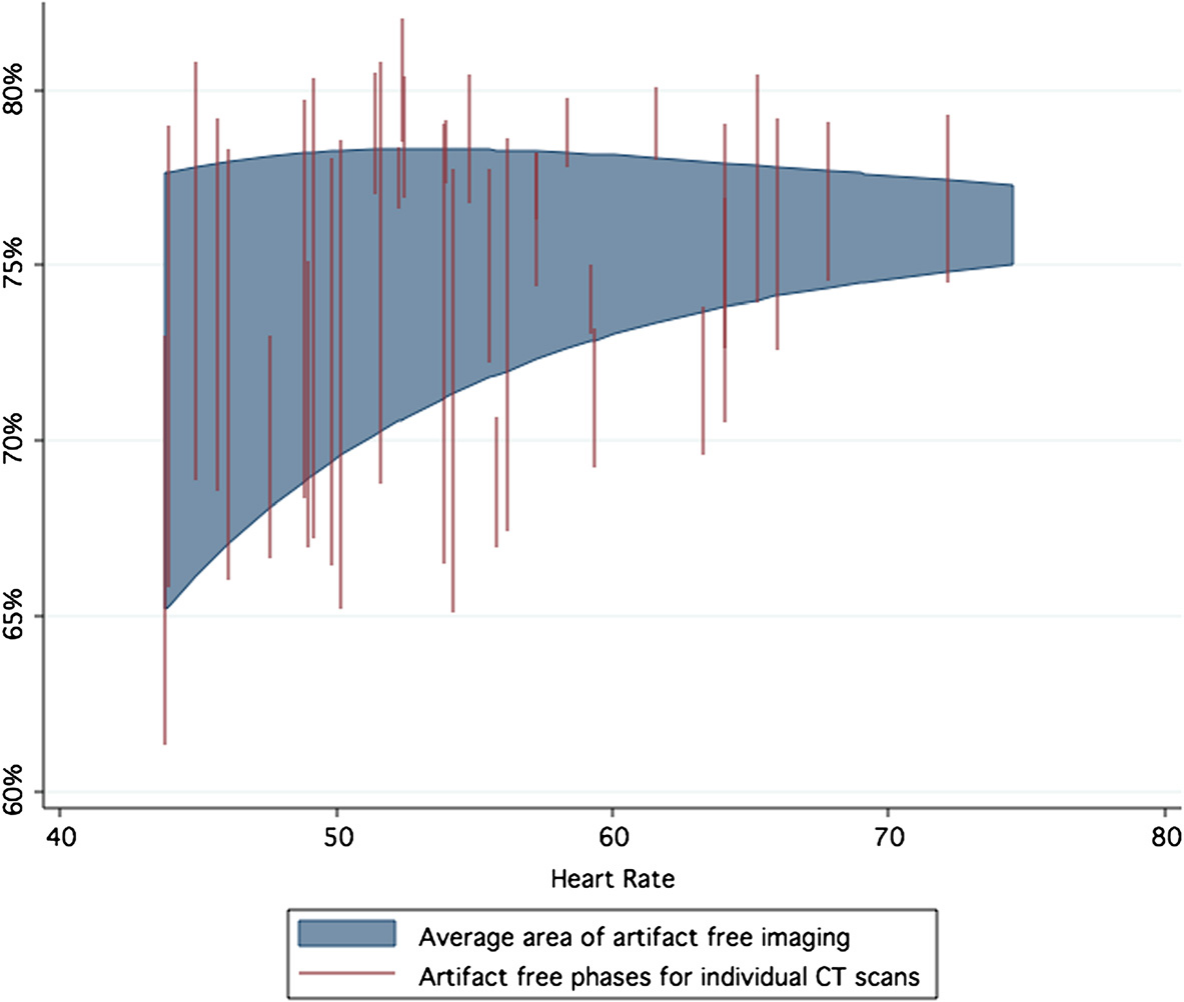
**Motion free-acquisition window for the right coronary artery from the CT validation set.** Very substantial variation in the motion-free period exists between individual studies. The acquisition window for the Right Coronary artery is relatively reduced and narrows more rapidly than the Circumflex artery.

While the heart rate guided phase target was statistically superior to the fixed target, both approaches demonstrated more motion artifact than could be predicted on the basis of coronary physiology alone (Figures 7 and 8). In 29 cases, no motion artifact was present in either artery at either the heart rate adjusted target or at the 75% phase. In 19 cases (38%), including 8 cases where no motion artifact free images occurred, image artifact was present with both timing strategies. The use of heart rate adjustment of CT timing would have led to the elimination of motion artifact in a net of 2 (4%) of patients within the validation cohort.

## Discussion

This study demonstrates the role of careful examination of the physiology of coronary motion in understanding the optimal timing of cardiac CT within diastole and the impact of image acquisition speed on motion artifact. The study also demonstrates the difficulties of cardiac imaging timing given the inherent physiological variability in coronary motion. While we primarily assessed the effects of coronary motion on cardiac CT, the research may also have a role in other gated imaging modalities such as cardiovascular MRI.

The temporal resolution of half-scan reconstruction of current generation single source cardiac CT lies between 140 and 175 ms. While current dual-source imaging reduces the temporal resolution to 70 ms-83 ms [1,13], in its lowest dose, high-pitch exemplification, the time taken to image the entire axial cardiac volume remains in the order of 280 ms. Retrospective gating of cardiac CT, and increasing the window of acquisition can go some way towards reducing the effects of cardiac motion at higher heart rates, but at the cost of greatly increased radiation dose. Retrospective gated cardiac CT may deliver 4 or 5 times the ionizing radiation of prospective CT [14] and even when prospective cardiac CT protocols are used, for every extra 100 ms of the cardiac cycle acquired, radiation dose is increased by up to 45% [15]. On the other hand, very low dose prospectively gated cardiac CT acquired during minimal phase acquisition enables only one or a very narrow band of phases to be analyzed, compromising diagnosis in the case of motion artifact. Accurate timing of cardiac CT is therefore vital for both radiation dose reduction and prevention of imaging artifacts.

High temporal resolution imaging of coronary artery motion reveals interesting insights about coronary motion, which may be useful for effective cardiac imaging. Previous measurements of coronary artery motion using electron beam CT [16,17] or coronary angiography [7] are inadequate for the precise definition of the cardiac rest period within mid-diastole. This is because the low temporal resolution of these techniques can only provide information as to which broad band of phase acquisition is most appropriate according to heart rate. The issue as to whether end-systole or diastole is superior at a given heart rate is clinically important and has been previously studied [3,4,18-20]. Likewise the interaction of gantry rotation time, heart rate and image artifact requires careful attention in order to optimize image quality [21,22]. The current research adds to these prior works by further elucidating the nature, effects and variability of coronary motion during the imaging phase of mid-diastole.

The equations and tables provided are intended as a guide for gated cardiac imaging across all CT types. They indicate the heart rates at which diastolic imaging becomes viable, the window available for imaging and the chance of motion artifact for given heart rates. In the validation cohort, a strategy of varying the targeted phase according to heart rate was statistically superior to a fixed percentage acquisition in terms of proximity to the ideal imaging phase, although the extent of benefit was small. These tools should prove useful for the achievement of high quality cardiac CT imaging. Nevertheless, there are several limitations to the formulas and conclusions provided and they should not be applied without consideration.

Firstly, it should be noted that even small differences in absolute timing make a large difference in the optimal phase selection. Variation can occur due to triggering parameters, QRS measurement and biological variability. Beat to beat variation of more than 50 ms can be expected at most heart rates and no system of CT triggering can ever account for the unpredictability associated with ectopic beats or atrial fibrillation. The wide variation in the ideal imaging target within the CT validation cohort may have reflected ectopy during the period of heart-rate assessment, although as we were only able to capture the two heartbeats immediately prior to imaging we were unable to assess the impact of ectopy in this study. While measurement of time intervals on tissue Doppler has generally good reliability [23], the use of a single measurement in our study should also be considered a limitation and may have added to variability of tissue Doppler measurements.

Secondly, the definition of motion artifact used in this study was conservative and it is likely that a degree of motion artifact may be tolerated without affecting clinical interpretability. The effect of motion artifact on diagnosis is inherently subjective and the precise heart-rate boundaries of CT interpretability may vary from the predictions of this study.

Lastly, the difference between a fixed 75% phase trigger and a heart rate adjusted strategy was small, and both strategies benefit from a degree of extra phase acquisition at most heart rates and gantry speeds. CT coronary angiography performed as a part of this study was designed to ensure coverage of mid-diastole and analysis of systolic or end-systolic phases could not be performed. Adjustment of the precise imaging target within mid-diastole should be a consideration secondary to the more fundamental issue of whether end-systolic or diastolic imaging is required.

## Conclusions

While the benefit of heart rate adjustment for coronary CT phase target selection within any individual patient is small, over a large cohort where dose minimization through single-phase acquisition is mandated, even minor adjustments in diastolic timing may result in a reduced chance of image motion artifact. Knowledge of physiological coronary motion reinforces the importance of heart rate control, acquisition time and phase selection in cardiac imaging.

## Abbreviations

LCX: Left circumflex artery; RCA: Right coronary artery; PMCM: Period of minimal cardiac motion; IVRT: Isovolumic relaxation time.

## Competing interests

The authors declare that they have no competing interests.

## Authors’ contributions

JO assisted with CT measurements, study design, data analysis and drafted the manuscript. JP performed CT and tissue Doppler measurements and critically edited the manuscript. MF participated in study conception and design. CYY critically edited the manuscript. NS critically edited the manuscript. JM participated in study design, conception, interpretation and helped draft the manuscript. All authors read and approved the final manuscript.

## Pre-publication history

The pre-publication history for this paper can be accessed here:

http://www.biomedcentral.com/1471-2342/13/5/prepub
